# S79. ENHANCING WORKING MEMORY IN SCHIZOPHRENIA USING 1MA AND 2MA TRANSCRANIAL DIRECT STIMULATION TO THE LEFT DORSOLATERAL PREFRONTAL CORTEX

**DOI:** 10.1093/schbul/sby018.866

**Published:** 2018-04-01

**Authors:** Irina Papazova, Wolfgang Strube, Benedikt Becker, Bettina Henning, Tobias Schwippel, Andreas Fallgatter, Frank Padberg, Ulrich Palm, Peter Falkai, Christian Plewnia, Alkomiet Hasan

**Affiliations:** 1 Ludwig Maximilian University Munich; 2 University Hospital Tuebingen; 3 University of Tuebingen

## Abstract

**Background:**

Cognitive impairment is a key symptom of schizophrenia, causing patients’ occupational disability and worsening their life quality. Yet, the treatment options are still scarce. Recent research suggests that transcranial direct stimulation (tDCS) to the dorsolateral prefrontal cortex (DLPFC) could enhance a crucial cognitive process such as working memory. Here, for the first time we examined the effects of tDCS on simultaneous working memory performance in schizophrenia patients in regard of stimulation intensity and cognitive load.

**Methods:**

Forty schizophrenia patients (N = 40) participated in two separate double-blind, sham-controlled experiments, both consisting of a pre-stimulation baseline, an active anodal and a sham tDCS single-session. Stimulation application was conducted to the F3 (anode) and to the right deltoid muscle (cathode) for 21 min. In Experiment 1 (N = 20) patients received tDCS at 1 mA and Experiment 2 (N = 20) – at 2 mA. In total, 120 experimental sessions were performed. Working memory was measured during stimulation using a verbal n-back task with three cognitive loads - 1-back, 2-back, 3-back. Applying the Signal Detection Theory, we estimated the discriminability index d prime, which together with reaction times served as study outcomes. Using several RM-ANOVAs we compared working memory performance during sham and active tDCS across all cognitive loads for each experiment. In a subsequent mixed-model RM-ANOVA, we pooled data from both experiments and analyzed differences in working memory performance in regard of stimulation intensity.

**Results:**

Data analysis showed significant greater d prime values during active tDCS than during sham tDCS only in Experiment 1 (F1, 19 = 4.48, p = .048). In Experiment 2, there was a numeric improvement of d prime during tDCS that however did not reach significance (F1, 19 = 2.31, p = .145). The subsequent mixed-model RM-ANOVA revealed a significant overall effect of brain stimulation, prompting higher d prime values (F1, 38 = 6.05, p = .019), but no main of stimulation intensity (p = .392). Analysis on reaction times revealed no significant results.

**Discussion:**

This is the first study comparing the online effects of 1mA and 2mA tDCS on working memory in schizophrenia patients. In line with previous research, tDCS improved working memory functioning in schizophrenia. However, this enhancement did not differ between stimulation intensities, implying that tDCS effects on cognition could be dose independent. Overall, our results provide further evidence that tDCS may be an effective and feasible intervention for cognitive impairment in schizophrenia and underline the need for future research on the specific stimulation parameters.

